# Assessment of Soybean Oil Oxidative Stability from Rapid Analysis of its Minor Component Profile

**DOI:** 10.3390/molecules25204860

**Published:** 2020-10-21

**Authors:** Ana S. Martin-Rubio, Patricia Sopelana, María D. Guillén

**Affiliations:** Food Technology, Faculty of Pharmacy, Lascaray Research Center, University of the Basque Country (UPV/EHU), 01006 Vitoria, Spain; anamaria.sanmartin@ehu.eus (A.S.M.-R.); patricia.sopelana@ehu.eus (P.S.)

**Keywords:** soybean oil, oxidative stability, ^1^H nuclear magnetic resonance, direct immersion SPME, tocols, sterols, free fatty acids, hydroperoxides, epoxides, aldehydes

## Abstract

The minor components of vegetable oils are important for their oxidative stability. In order to know to what extent they can influence oil behaviour under oxidative conditions, two commercial soybean oils, one virgin and the other refined, both with very similar compositions in acyl groups but differing in their minor component profiles, were subjected to accelerated storage conditions. They were characterized by ^1^H nuclear magnetic resonance (NMR) and direct immersion solid-phase microextraction coupled to gas chromatography/mass spectrometry (DI-SPME-GC/MS), while oil oxidation was monitored by ^1^H-NMR. The lower levels of tocols and sterols in the virgin oil, together with its higher free fatty acid content when compared to the refined one, result in a lower oxidative stability. This is deduced from faster degradation of acyl groups and earlier generation of hydroperoxides, epoxides, and aldehydes in the virgin oil. These findings reveal that commercial virgin soybean oil quality is not necessarily higher than that of the refined type, and that a simple and rapid analysis of oil minor components by DI-SPME-GC/MS would enable one to establish quality levels within oils originating from the same plant species and similar unsaturation level regarding composition in potentially bioactive compounds and oxidative stability.

## 1. Introduction

It is known that the oxidative stability of oils depends on their unsaturation degree, determined by their acyl group profile [1], but also on their composition in minor components [2,3]. These minor oil components include several types of compounds with attributed antioxidant ability like tocopherols and tocotrienols, squalene, sterols, or cyclic dipeptides [4,5,6,7], but also other compounds reported to increase the susceptibility of oils to oxidation, such as free fatty acids [2]. In this context, it must be noticed that although most seed oils are usually subjected to a refining process, some can also be consumed without being refined, which is to say as either virgin or cold-pressed oils. Therefore, bearing in mind that oil refining processes can reduce the concentration of minor components like tocopherols, carotenoids, or free fatty acids [8], it is possible to find commercial vegetable oils originating from the same plant species with very similar compositions in main components (acyl groups) but which differ in minor ones. Despite this, the possibility of considering the minor component profile as an indicator of the oxidative stability of edible oils seems to have been little exploited. Regarding this issue, some studies have been conducted in recent years aimed at finding relationships between the composition of a wide range of vegetable oils and their resistance to oxidation [9,10,11,12,13,14]. In these works, in general, attention is paid to both fatty acid composition and minor components like polyphenols, sterols, tocols, β-carotene, lutein, or chlorophyll. However, in two of them [13,14], the composition in main components was not analyzed, even though this also affects the oxidative stability of oils [1]. Another subject that should be taken into account when analyzing the conclusions drawn from the aforementioned studies is the selection of the Rancimat test, performed at temperatures of either 110 or 120 °C, to assess oil oxidative stability. Thus, although according to Farhoosh and Moosavi [15], it can be useful to act as a “screening” test to identify oils with lower stability under frying conditions, the only oxidation products considered are volatile organic acids; this represents an extreme over-simplification of the oxidation process, during which myriads of compounds, many of them non-volatile, can be generated [16]. Moreover, when performing oxidative stability tests at high temperatures, it must also be taken into account that the reactions occurring under these conditions are different from those occurring at lower temperatures [3], such as during the storage of oils, and that the effect of antioxidants can also vary depending on temperature [17]. Indeed, measurement of the induction period using a Rancimat apparatus led to surprising conclusions concerning the stability of corn and soybean oils [11], which was reported to be similar to that found by other researchers for cold-pressed olive oil [18], even though the oxidative stability of the latter has been shown to be considerably higher than that of the former, both under accelerated storage conditions [19,20] and at frying temperatures [21].

In light of all the above, this work aimed to study the influence of the composition in minor components of two commercially available soybean oils, one virgin and the other refined, on their oxidative stability under accelerated storage conditions considering the whole oxidation process. The composition in acyl groups and the unsaturation degree of the two selected oils were analyzed by ^1^H nuclear magnetic resonance (^1^H-NMR), while the study of the minor oil component profile was accomplished by means of direct immersion solid-phase microextraction followed by gas chromatography/mass spectrometry (DI-SPME-GC/MS). This methodology has the extraordinary advantage of providing information about different groups of minor components simultaneously and rapidly in comparison with the methodologies usually employed for this purpose, with no modification of the sample [22]. Next, the oils the subject of study were submitted to an accelerated storage (AS) process at 70 °C and their respective behaviors were studied by means of ^1^H-NMR, which, unlike the methodologies used in the aforementioned studies, provides a global view of the oxidation process. To this aim, the evolution throughout the AS process of the different types of oil acyl groups and the generation of various kinds of oxidation products, which can be considered representative enough to achieve the main goal of this work (hydroperoxides, epoxides, and aldehydes), were monitored.

## 2. Results and Discussion

### 2.1. Characterization of the Commercial Virgin and Refined Soybean Oils

#### 2.1.1. Composition in Main Components Determined by ^1^H-NMR

The samples subject of study were two commercial soybean oils from Spanish companies, one of them virgin (VSO: Virgin Soybean Oil), and the other one refined (RSO: Refined Soybean Oil), from different producers. The molar percentages of the different kinds of acyl groups in the two types of soybean oil are presented in Table 1.

These data reveal that both types of soybean oils display very similar proportions of acyl groups, and the same can be said of their unsaturation degree, estimated from the ratio of unsaturated to saturated protons (see Section 3.3.3), which was found to be 1 to 10.03 for VSO and 1 to 9.94 in RSO. Therefore, it seems reasonable to assume that the differences in their respective oxidative stabilities will be due to their composition in minor components.

#### 2.1.2. Composition in Minor Components Determined by DI-SPME-GC/MS

**Figure 1:**
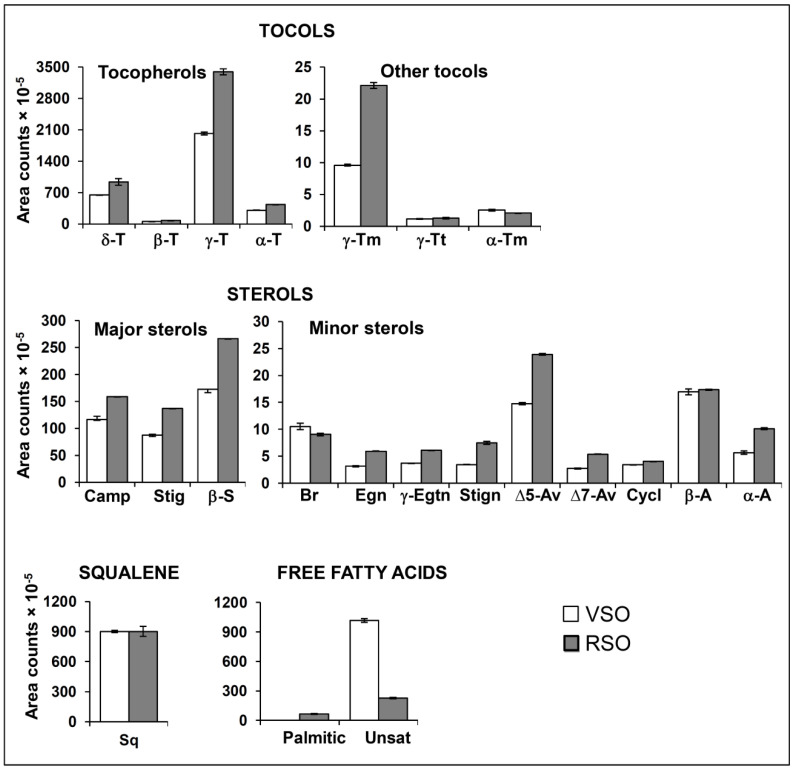
Bar graphics representing the abundance, expressed as arbitrary area units of the mass spectrum base peak of each compound (see Table S1, Supplementary Material) extracted from the total ion chromatograms obtained by DI-SPME/GC-MS, divided by 10^5^, in VSO and RSO of: tocols (tocopherols and others), sterols, squalene, and free fatty acids. All the figures reported are mean values corresponding to 2 replicates. T: tocopherol; Tm: tocomonoenol; Tt: tocotrienol; Camp: campesterol; Stig: stigmasterol; S: sitosterol; Br: brassicasterol; Egn: ergostanol; Egtn: ergostenol; Stign: stigmastanol; Av: avenasterol; Cycl: cycloartenol; A: amyrin; Sq: squalene; Unsat: sum of oleic, linoleic, and linolenic acids.

The methodology used here for the determination of minor oil components allows one to compare the amounts and relative proportions of several types of compounds in different oils. Figure 1 shows the abundances of the main minor components that are expected to influence the oxidative stability of soybean oil. These include compounds with attributed antioxidant ability like tocopherols (α-, β-, γ-, and δ-) and other tocols, squalene, and sterols [5,6,7], but also free fatty acids, which could favor lipid oxidation [2].

It can be observed that, contrary to what might be expected bearing in mind the denomination of the studied oils, the refined one (RSO) exhibits a higher level of tocols, among which tocopherols account by far for the highest proportion. Regarding this issue, it should be noticed that lower levels of tocols and of other types of compounds with antioxidant ability were found in another virgin soybean oil studied previously in comparison with the refined one [23], and that other authors also reported a higher concentration of tocopherols in commercial refined sunflower oil than in another virgin cold-pressed oil originating from the same plant species [12]. Nonetheless, the tocopherol profile is the same in both cases, with γ-tocopherol being the most abundant, followed by δ- and α-tocopherols, in line with data from other studies [22,24]. It is worth noticing the presence in both oils of γ-tocotrienol, also with an attributed antioxidant ability [6] and not detected in other studies concerning soybean oil [24,25], as well as of γ- and α-tocomonoenols; the latter type of compounds might also act as antioxidants taking into account findings relative to the δ-isomer [26]. Other phenolic compounds different from tocols, considered important antioxidants in other plant-derived foodstuffs, are in low concentration in soybean oil [27]. Therefore, although data about them are not provided, it is not expected that they greatly affect the oxidative stability of this type of oil.

Regarding sterol content, which includes desmethylsterols but also less commonly found 4,4′-dimethyl sterols like α- and β-amyrin, this is also higher in RSO than in VSO, β-sitosterol being the most abundant, in agreement with the findings of other authors [28]. The squalene concentration, instead, is basically the same in both oils. Despite these results appearing somewhat surprising considering that refining of soybean oil depletes the level of certain minor components [8], it must be taken into account that several factors other than the refining process can also affect the minor oil component composition of commercial oils from different suppliers. Thus, as pointed out by Chu and Lin [29], the tocopherol content of soybean oil can also be influenced by the state of the soybeans or the proportion of damaged beans, together with storage conditions and length. In addition, soybean variety and climatic factors can also affect soybean oil composition [30].

By contrast, Figure 1 shows that the amount of free fatty acids, both saturated and unsaturated, is higher in VSO than in RSO, which is in agreement with the removal of this type of minor oil component during the refining process [8].

### 2.2. Study by ^1^H-NMR of the Evolution Under AS Conditions of VSO and RSO 

**Figure 2:**
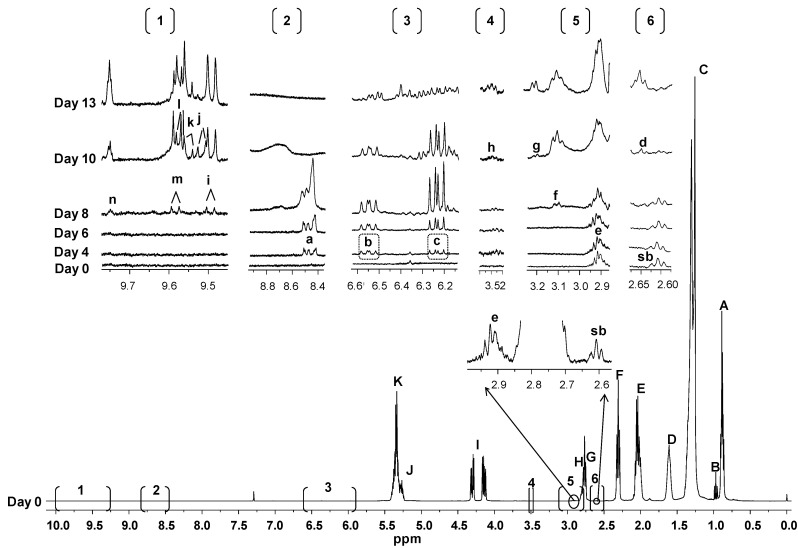
^1^H-NMR spectrum of sample RSO before being subjected to the AS process, together with the enlargements of some spectral regions where changes occur throughout time. Letters agree with those in Table S2 (Supplementary Material), considering that “e” includes signals “e1–e6”, “f” signals “f1–f3”, and “h” signals “h1+h2”. The plots corresponding to the same ^1^H-NMR spectral region are presented at a fixed value of absolute intensity, for them to be valid for comparative purposes. “sb”: side band of the *bis*-allylic protons signals (H+G).

As stated above, in order to assess the influence of the composition in minor components on the oxidation process of the studied oils, the degradation of the various types of oil acyl groups, together with the generation of different groups of oxidation products, were monitored by ^1^H-NMR. The evolution of some spectral regions where changes occurred throughout the AS process of RSO can be observed in Figure 2, and a similar figure showing this same information for the VSO sample is included in the Supplementary Material (see Figure S1).

#### 2.2.1. Evolution of the Different Types of Oil Acyl Groups

**Figure 3:**
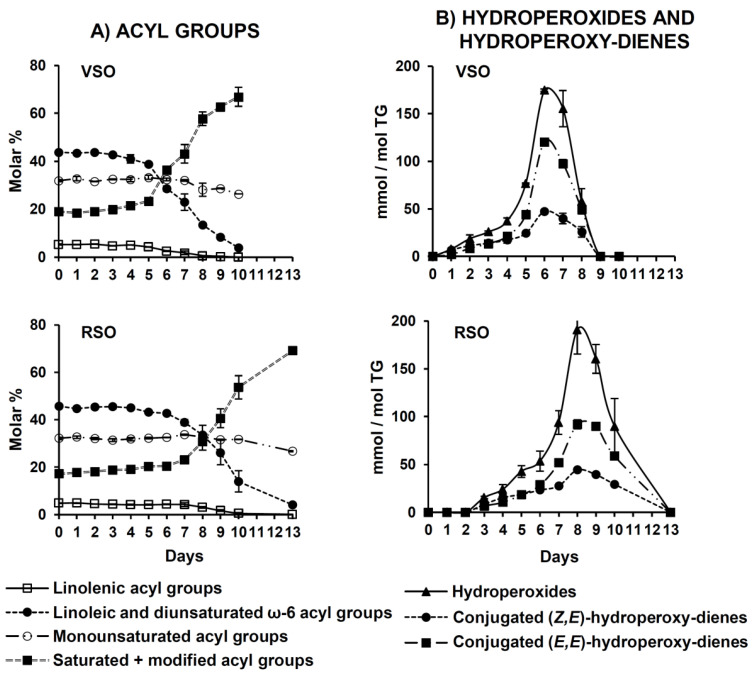
Evolution throughout the AS process in VSO and RSO of: (**A**) the molar percentages of linolenic, linoleic, and diunsaturated ω-6, monounsaturated, and saturated+modified acyl groups; and (**B**) the concentrations, in mmol/mol TG (triglycerides), of hydroperoxides and of their associated conjugated (*Z*,*E*)- and (*E*,*E*)-dienes. All the figures reported are mean values corresponding to 2 replicates.

The evolutions of the different kinds of oil acyl groups, expressed in molar percentages, are represented versus time in days in Figure 3A. This graph shows that the molar percentages of all the types of unsaturated groups, especially those of the polyunsaturated ones (linolenic and linoleic), decrease with time both in VSO and in RSO, with this diminution being slow during a first stage but very quick afterwards. In consequence, the molar percentage of saturated + modified (S + M) groups increases accordingly. When comparing the evolution of the two studied soybean oils, it is observed that the first phase of acyl group degradation is longer in RSO than in VSO (7 and 5 days, respectively), which results in a slower degradation of the former and in a longer total oil polymerization process (13 days against 10 in VSO).

#### 2.2.2. Formation and Evolution of Oxidation Products

- Hydroperoxides giving signal between 8.3 and 9 ppm, and their associated conjugated (*Z*,*E*)- and (*E*,*E*)-dienes

The evolution of the concentration of total hydroperoxides, expressed in millimoles per mole of triglyceride (mmol/mol TG), can be estimated from signal “a” (see Table S2 and Figure 2) and is shown in Figure 3B. The first noticeable feature is that in VSO, hydroperoxides were already detectable after the first day under AS conditions, while in RSO these products were not observed until day 3, indicating lower oxidative stability of the virgin oil. In line with acyl group evolution, the hydroperoxide concentration increased slowly over 4 and 6 days in VSO and RSO, respectively, but then the growing rate became much higher until the maximum was reached after 6 days in VSO and 8 days in RSO. All this confirms that oxidation proceeds faster in VSO than in RSO.

Hydroperoxides generated in oils under AS conditions usually support conjugated diene systems with either (*Z*,*E*)- or (*E*,*E*)-isomerism, which can be monitored separately by measuring their corresponding signals (see Table S2, signals “b” and “c”, respectively). The evolution of these signals throughout the AS process can be observed in Figure 2, while the progress of their estimated concentrations in the two studied oils is shown in Figure 3B. The latter reveals that, in line with the total hydroperoxide evolution, the emergence of both types of hydroperoxy-dienes occurs earlier in VSO than in RSO; notwithstanding, their progress over time exhibits the same trend in both oils.

- Epoxides

Epoxides constitute a relevant group of secondary lipid oxidation products, due to their notable concentration and to their potential toxicity and other adverse effects [31], despite which they have received little attention in oxidation studies.

**Figure 4:**
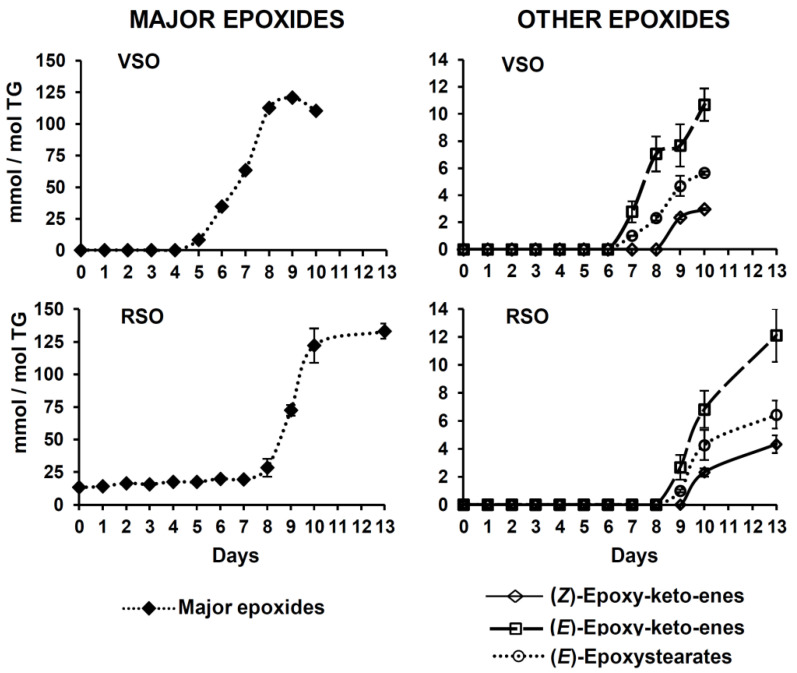
Evolution throughout the AS process of the concentrations, in mmol/mol TG, of different types of epoxides in VSO and RSO. All the figures reported are mean values corresponding to 2 replicates.

Table S2, which compiles some of these compounds, shows that only some of them, like (*E*)-epoxystearates (letter “d”) or (*E*)-epoxy-keto-enes (letters “g” and “h”), generate signals isolated from those of the rest of epoxides. However, an estimation of their overall amount can be made, which can be useful to assess the contribution of this type of oxidation product to the total compounds generated. The evolution of their corresponding signals with time can be observed in Figure 2 (letters “d”, “e”, “f”, “g”, and “h”), while the progress of their estimated concentrations throughout the AS process is displayed in Figure 4, in mmol/mol TG. As pointed out in the Supplementary Material, most of the epoxides considered, designated as “major epoxides”, give their signals between 2.87 and 3.17 ppm approximately (see Table S2 and Figure 2). According to the data in Table S2, they are supposed to include (*Z*)-epoxystearates derived from oleic groups, as well as different types of epoxy-compounds coming from polyunsaturated groups, some of them supporting other functional groups like, for example, hydroperoxy- or hydroxy-ones.

With regard to major epoxides, it is worth noticing that a part of this type of epoxides, presumably monoepoxides from linolenic and/or linoleic groups, was present in the fresh refined soybean oil, before being subjected to the heating process. This is deduced from the observation of signal “e” in Figure 2 (see the spectrum corresponding to day 0), which in the absence of epoxides should resemble that of the right side-band of *bis*-allylic protons signal (see signal “sb”). However, in the case of RSO, it seems clear that there are additional compounds contributing to signal “e”. Figure 4 reveals that, as might be expected from the evolution of hydroperoxides (see Figure 3B), the appearance of major epoxides in VSO occurs before their concentration begins to increase in RSO.

Apart from the aforementioned major epoxides, other types of epoxy-compounds are also generated throughout the oil oxidation process, although in considerably lower concentrations: epoxy-keto-enes and (*E*)-epoxystearates. These derive from the degradation of polyunsaturated and oleic groups, respectively, and their corresponding evolutions are also shown in Figure 4. As in the case of major epoxides, both types of compounds are detected later in RSO than in VSO, their evolution with time being similar in the two studied oils.

- Aldehydes

**Figure 5:**
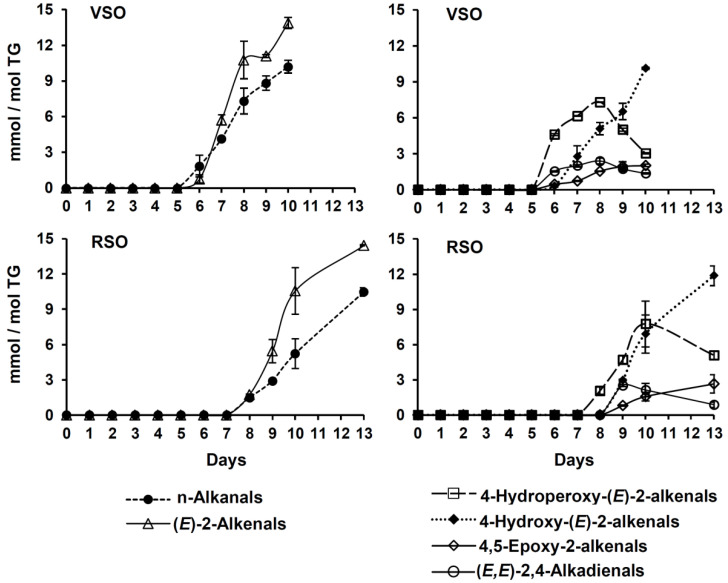
Evolution throughout the AS process of the concentrations, in mmol/mol TG, of the different types of aldehydes in VSO and RSO. All the figures reported are mean values corresponding to 2 replicates.

Due to the reactivity and toxicity of some aldehydes, such as the oxygenated α,β-unsaturated ones [32], this type of compound constitutes an important group of oxidation products. However, the estimation of their concentration is usually based on the determination of only a few of them, with malondialdehyde, measured through the TBARS (thiobarbituric acid reactive substances) assay, being the sole target of many studies [33]. The progress of the ^1^H-NMR signals of the different kinds of aldehydes throughout the AS process in the refined oil can be observed in Figure 2 (signals “i” to “n”), and the evolutions of their respective concentrations in the two studied oils in Figure 5.

As shown in Figure 5, aldehydes, including the most toxic ones (4-hydroperoxy-(*E*)-2-alkenals, 4-hydroxy-(*E*)-2-alkenals and 4,5-epoxy-2-alkenals), are also generated earlier in VSO than in RSO (after 6 and 8 days under AS conditions, respectively). Moreover, while all the different kinds of aldehydes are detected at the same time in VSO, the aldehyde appearance process in RSO is staggered, in such a way that 4-hydroxy-(*E*)-2-alkenals, 4,5-epoxy-2-alkenals and (*E*,*E*)-2,4-alkadienals emerge one day later than the first ones detected (n-alkanals, (*E*)-2-alkenals, and 4-hydroperoxy-(*E*)-2-alkenals). This indicates a lower generation rate of this type of oxidation product in RSO. With regard to the evolution with time of the several groups of aldehydes, this is the same in both oils.

### 2.3. Final Remarks

All the results reported above evidence a lower oxidative stability of the virgin soybean oil when compared to the refined one. This agrees with the findings of some authors who have also shown increased resistance to oxidation for commercial refined oils than for virgin cold-pressed ones originating from the same plant species, on the basis of the Rancimat method [12]. However, this should not be linked to the designation of the oil as either virgin or refined, but to their composition, which could be influenced, as abovementioned, by various factors related both to the raw material and to the oil processing operations. All these variables can differ from one oil to another, especially if they come from different manufacturers.

It is also worth noticing that, although a higher concentration of tocopherols seems to contribute to a higher oxidative stability in the soybean oils here studied, this relationship should not be directly extrapolated to other types of vegetable oils where α-tocopherol and not γ-tocopherol was the major one. This assertion is based on the conclusions of many works, which suggest that increasing concentrations of α-tocopherol might result in a lower oil oxidative stability [17,34]. Furthermore, Zaunschirm and coworkers [35] reported that more elevated ratios between the sum of γ- and δ-tocopherol concentrations and the concentration of α-tocopherol seemed to be related to a lower oxidation rate.

## 3. Materials and Methods

### 3.1. Characterization of the Oils Subject of Study

#### 3.1.1. Analysis of the Main Oil Components (Acyl Groups)

The molar percentages of the different types of oil acyl groups were determined by ^1^H-NMR, as in previous works [36], by means of the equations given in the Supplementary Material (S1–S4).

#### 3.1.2. Analysis of the Minor Oil Components

Extraction of the minor oil components was performed by means of DI-SPME-GC/MS, according to the methodology described by Alberdi-Cedeño and coworkers [22]. This involves the immersion of a fiber of 65 μm StableFlex polydimethylsiloxane/divinylbenzene (PDMS/DVB), acquired from Supelco (Bellefonte, PA, USA), into 6 mL of edible oil at room temperature for 45 min. The thermal desorption of the extracted compounds and their subsequent separation was carried out in a gas chromatograph equipped with a mass spectrometry detector (Agilent Technologies, Santa Clara, CA, USA) as described in the above-mentioned work. The analysis was carried out in duplicate.

Identification and semi-quantification of the extracted components were accomplished as described in the Supplementary Material.

### 3.2. Accelerated Storage (AS) Process

First, 10-g portions of each oil sample were poured into plastic Petri dishes of 80 mm diameter for each of the days monitored throughout the AS process. These were heated at 70 °C in a convection oven with circulating air but without forced convection, simulating AS conditions. The evolution of the samples was followed by ^1^H-NMR until their total polymerization. The AS process was carried out in duplicate with the two studied samples.

### 3.3. Monitoring by ^1^H-NMR of the Evolution of VSO and RSO Throughout the AS Process

#### 3.3.1. Operating Conditions

The ^1^H-NMR spectra of all the samples taken throughout the AS process were acquired using a Bruker Avance (Rheinstetten, Germany) 400 spectrometer operating at 400 MHz, with the weight of each sample being approximately 0.16 g. These were mixed in a 5-mm diameter tube with 400 µL of deuterated chloroform containing 0.2% of non deuterated chloroform and a small amount (0.03%) of tetramethylsilane as the internal reference. The acquisition parameters used and the experimental conditions were the same as in previous works [36].

#### 3.3.2. Identification of Some Components

The identification of the products formed throughout the AS process was carried out on the basis of the signal assignment shown in Table S2, made from bibliographic data and with the aid of several standard compounds, also given in the Supplementary Material.

#### 3.3.3. Quantitative Data Derived from ^1^H-NMR Spectra

Equations (S1)–(S4) were also used to estimate the molar percentages of several kinds of oil acyl groups throughout the AS process.

In addition, the unsaturation degree of the studied oils was determined from the area of olefinic protons (signal “K” in Table S2) relative to that of the sum of all kinds of saturated protons (signals “A”–“H”, both included, in Table S2).

The concentrations of the different types of oxidation products were estimated as mmol/mol TG as described in the Supplementary Material, using Equation (S5).

## 4. Conclusions

The findings of this study reveal to what extent the composition in minor components of two commercial soybean oils with very similar compositions in acyl groups can affect their oxidative stability and evolution under AS conditions. Thus, oxidation proceeds more slowly in the refined oil, which is the one exhibiting the highest concentrations of tocols and sterols, and the poorest in free fatty acids. This is deduced from a slower degradation of oil acyl groups and from a later and, in some cases, more gradual generation of oxidation products.

Contrary to what the denomination of the two soybean oils might suggest, the virgin one showed lower tocopherol and sterol contents. Therefore, it should not be generally assumed that the concentration of virgin soybean oils in minor components considered to be beneficial for human health is higher than that of the refined ones.

Finally, the main outcome of this work is that a simple analysis of the composition in minor components of commercial oils by means of direct immersion SPME followed by GC/MS would make it possible to establish different categories or quality levels within oils originating from the same plant species, in terms of not only the composition in potentially bioactive compounds but also of oxidative stability. This latter parameter is crucial for the safety of oils, since it directly affects the generation rate of toxic oxidation products. Thus, the establishment of parameters indicative of this quality, not considered until now, might be valuable for oil producers, who could add value to their products, for consumers and also for the food industry, which would have a means to identify oils with different oxidative stabilities.

## Figures and Tables

**Table 1:** Molar percentages of the main types of acyl groups in each of the studied oils.

Sample	Linolenic	Linoleic	Oleic	Saturated
VSO	5.3 ± 0.7	43.7 ± 0.5	31.9 ± 0.7	19.1 ± 0.5
RSO	4.8 ± 0.1	45.8 ± 0.8	32.2 ± 0.5	17.2 ± 1.2

## Supplementary Materials

The following are available online. Table S1: Minor components found in the soybean oils studied by means of DI-SPME-GC/MS analysis, together with their respective molecular weight (MW) and their mass spectra base peaks (BP), Figure S1: ^1^H-NMR spectrum of sample VSO before being subjected to the AS process, together with the enlargements of some spectral regions where changes occur throughout time, Table S2: Chemical shifts, multiplicities and assignments of the ^1^H-NMR signals in CDCl3 of the main types of triglyceride (TG) protons, and of some oxidation compounds, present in the different soybean oil samples, before and throughout the oxidation process, Equations used for the determination, from ^1^H-NMR spectral data, of the molar percentages of the different kinds of oil acyl groups and of the concentrations of the various kinds of oxidation products monitored throughout the AS process, Procedure for the identification and semi-quantification of some compounds by GC/MS, Standard compounds used for the identification of some compounds by ^1^H-NMR.

## References

[1] Martínez-Yusta A., Goicoechea E., Guillén M.D. (2014). A review of thermo-oxidative degradation of food lipids studied by ^1^H-NMR spectroscopy: Influence of degradative conditions and food lipid nature. Compr. Rev. Food Sci. Food Saf..

[2] Choe E., Min D.B. (2006). Mechanisms and factors for edible oil oxidation. Compr. Rev. Food Sci. Saf..

[3] Kamal-Eldin A. (2006). Effect of fatty acids and tocopherols on the oxidative stability of vegetable oils. Eur. J. Lipid Sci. Technol..

[4] Alberdi-Cedeño J., Ibargoitia M.L., Guillén M.D. (2017). Bioactive compounds detected for the first time in corn oil: Cyclic dipeptides and other nitrogenated compounds. J. Food Compost. Anal..

[5] Dessi M.A., Deiana M., Day B.W., Rosa A., Banni S., Corongiu F.P. (2002). Oxidative stability of polyunsaturated fatty acids: Effect of squalene. Eur. J. Lipid Sci. Technol..

[6] Seppanen C.M., Song Q., Csallany A.S. (2010). The antioxidant functions of tocopherol and tocotrienol homologues in oils, fats, and food systems. J. Am. Oil Chem. Soc..

[7] Yoshida Y., Niki E. (2003). Antioxidant effects of phytosterol and its components. J. Nutr. Sci. Vitaminol..

[8] Jung M.Y., Yoon S.H., Min D.B. (1989). Effects of processing steps on the contents of minor compounds and oxidation of soybean oil. J. Am. Oil. Chem. Soc..

[9] Ayyildiz H.F., Topkafa M., Kara H., Sherazi S.T.H. (2015). Evaluation of fatty acid composition, tocols profile, and oxidative stability of some fully refined edible oils. Int. J. Food Prop..

[10] Bozan B., Temelli F. (2008). Chemical composition and oxidative stability of flax, safflower and poppy seed and seed oils. Bioresour. Technol..

[11] Castelo-Branco V.N., Santana I., Di-Sarli V.O., Freitas S.P., Torres A.G. (2016). Antioxidant capacity is a surrogate measure of the quality and stability of vegetable oils. Eur. J. Lipid Sci. Technol..

[12] Redondo-Cuevas L., Castellano G., Torrens F., Raikos V. (2018). Revealing the relationship between vegetable oil composition and oxidative stability: A multifactorial approach. J. Food Compost. Anal..

[13] Yang M., Zheng C., Zhou Q., Huang F., Liu C., Wang H. (2013). Minor components and oxidative stability of cold-pressed oil from rapeseed cultivars in China. J. Food Compost. Anal..

[14] Zheng C., Yang M., Zhou Q., Huang F., Li W., Liu C. (2018). Bioactive compounds and antioxidant activities of cold-pressed seed oils. Oil Crop. Sci..

[15] Farhoosh R., Moosavi S.M.R. (2007). Rancimat test for the assessment of used frying oils quality. J. Food Lipids.

[16] Frankel E.N., Neff W.E., Rohwedder W.K., Khambay B.P., Garwood R.F., Weedon B.C.L. (1977). Analysis of autoxidized fats by gas chromatography-mass spectrometry: II. Methyl linoleate. Lipids.

[17] Mancebo-Campos V., Salvador M.D., Fregapane G. (2014). Antioxidant capacity of individual and combined virgin olive oil minor compounds evaluated at mild temperature (25 and 40 °C) as compared to accelerated and antiradical assays. Food Chem..

[18] Läubi M.W., Bruttel P.A. (1986). Determination of oxidative stability of fats and oils: Comparison between the active oxygen method (AOCS Cd 12-57) and the Rancimat method. J. Am. Oil Chem. Soc..

[19] Guillén M.D., Ruiz A. (2005). Oxidation process of oils with high content of linoleic acyl groups and formation of toxic hydroperoxy- and hydroxyalkenals. A study by ^1^H nuclear magnetic resonance. A study by 1H nuclear magnetic resonance. J. Sci. Food Agric..

[20] Guillén M.D., Ruiz A. (2005). Study by proton nuclear magnetic resonance of the thermal oxidation of oils rich in oleic acyl groups. J. Am. Oil Chem. Soc..

[21] Guillén M.D., Ruiz A. (2008). Monitoring of heat-induced degradation of edible oils by proton NMR. Eur. J. Lipid Sci. Technol..

[22] Alberdi-Cedeño J., Ibargoitia M.L., Cristillo G., Sopelana P., Guillén M.D. (2017). A new methodology capable of characterizing most volatile and less volatile minor edible oils components in a single chromatographic run without solvents or reagents. Detection of new components. Food Chem..

[23] Martin-Rubio A.S., Ibargoitia M.L., Sopelana P., Guillén M.D. Controversia en relación al contenido de aceite de soja virgen y refinado en componentes beneficiosos para la salud. Proceedings of the 6th International Congress about Own-Checks and Food Safety.

[24] Cerretani L., Lerma-García M.J., Herrero-Martínez J.M., Gallina-Toschi T., Simó-Alfons E.F. (2009). Determination of tocopherols and tocotrienols in vegetable oils by nanoliquid chromatography with ultraviolet-visible detection using a silica monolithic column. J. Agric. Food Chem..

[25] Rao M.K., Perkins E.G. (1972). Identification and estimation of tocopherols and tocotrienols in vegetable oils using gas chromatography-mass spectrometry. J. Agric. Food Chem..

[26] Fiorentino A., Mastellone C., D’Abrosca B., Pacifico S., Scognamiglio M., Cefarelli G., Caputo R., Monaco P. (2009). δ-Tocomonoenol: A new vitamin E from kiwi (*Actinidia chinensis*) fruits. Food Chem..

[27] Siger A., Nogala-Kalucka M., Lampart-Szczapa E. (2008). The content and antioxidant activity of phenolic compounds in cold-pressed plant oils. J. Food Lipids.

[28] Phillips K.M., Ruggio D.M., Toivo J.I., Swank M.A., Simpkins A.H. (2002). Free and esterified sterol composition of edible oils and fats. J. Food Compost. Anal..

[29] Chu Y.H., Lin J.Y. (1993). Factors affecting the content of tocopherol in soybean oil. J. Am. Oil Chem. Soc..

[30] Erickson D.R. (2015). Practical Handbook of Soybean Processing and Utilization.

[31] Alberdi-Cedeño J., Ibargoitia M.L., Guillén M.D. (2020). Oxylipins associated to current diseases detected for the first time in the oxidation of corn oil as a model system of oils rich in omega-6 polyunsaturated groups. A global, broad and in-depth study by ^1^H-NMR spectroscopy. Antioxidants.

[32] Guillén M.D., Goicoechea E. (2008). Toxic oxygenated α,β-unsaturated aldehydes and their study in foods: A review. Crit. Rev. Food Sci. Nutr..

[33] Barriuso B., Astiasarán I., Ansorena D. (2013). A review of analytical methods measuring lipid oxidation status in foods: A challenging task. Eur. Food Res. Technol..

[34] Dolde D., Wang T. (2011). Oxidation of corn oils with spiked tocols. J. Am. Oil Chem. Soc..

[35] Zaunschirm M., Pignitter M., Kienesberger J., Hernler N., Riegger C., Eggersdorfer M., Somoza V. (2018). Contribution of the ratio of tocopherol homologs to the oxidative stability of commercial vegetable oils. Molecules.

[36] Guillén M.D., Uriarte P.S. (2012). Study by ^1^H-NMR spectroscopy of the evolution of extra virgin olive oil composition submitted to frying temperature in an industrial fryer for a prolonged period of time. Food Chem..

