# Supplementary material for: Assessment of Soybean Oil Oxidative Stability from Rapid Analysis of its Minor Component Profile
Source: Molecules. 2020 Oct 21;25(20):4860. doi: 10.3390/molecules25204860 (PMC7594062; doi:10.3390/molecules25204860)
Supplement: Supplementary file 1 [file molecules-25-04860-s001.zip › molecules-968551-supplementary.docx]

SUPPLEMENTARY MATERIAL

Assessment of Soybean Oil Oxidative Stability from Rapid Analysis of its Minor Component Profile

Ana S. Martin-Rubio; P. Sopelana; María D. Guillén*

Food Technology, Faculty of Pharmacy, Lascaray Research Center, University of the Basque Country (UPV/EHU), 01006 Vitoria, Spain; anamaria.sanmartin@ehu.eus (A.S.M.-R.); patricia.sopelana@ehu.eus (P.S.)

***** Correspondence: mariadolores.guillen@ehu.eus; Tel.: +34-945-013-081; Fax: 34-945-013014

Academic Editor: Jih-Jung Chen

Received: 30 September 2020; Accepted: 19 October 2020; Published: date

**Table S1.** Minor components found in the soybean oils studied by means of DI-SPME-GC/MS analysis, together with their respective molecular weight (MW) and their mass spectra base peaks (BPs). The asterisked compounds were acquired commercially and used as standards for identification purposes.

| **Compound** | **MW** | **BP** |
| --- | --- | --- |
| *Tocols* |  |  |
| δ-Tocopherol * | 402 | 402 |
| β-Tocopherol * | 416 | 416 |
| γ-Tocopherol * | 416 | 416 |
| α-Tocopherol * | 430 | 165 |
| γ-Tocomonoenol | 414 | 414 |
| α-Tocomonoenol | 428 | 428 |
| γ-Tocotrienol * | 410 | 151 |
|  |  |  |
| *Sterols* |  |  |
| Brassicasterol * | 398 | 314 |
| Campesterol * | 400 | 400 |
| Ergostanol | 402 | 215 |
| Stigmasterol * | 412 | 412 |
| γ-Ergostenol | 400 | 400 |
| β-Sitosterol * | 414 | 414 |
| Stigmastanol * | 416 | 416 |
| Δ5-Avenasterol * | 412 | 314 |
| Δ7-Avenasterol | 412 | 285 |
| Cycloartenol | 426 | 69 |
| β-Amyrin | 426 | 218 |
| α-Amyrin | 426 | 218 |
|  |  |  |
| *Squalene*  * | 410 | 69 |
|  |  |  |
| *Free fatty acids* |  |  |
| Palmitic acid * | 256 | 73 |
| Linoleic acid * | 280 | 67§ |
| Oleic Acid * | 282 | 55§ |
| Linolenic acid * | 278 | 79§ |

§Linoleic, oleic and linolenic acids mass spectra base peaks overlapped in the chromatograms. Thus, in order to quantify these compounds altogether, ion 55 was taken, since it is common to all these unsaturated fatty acids.


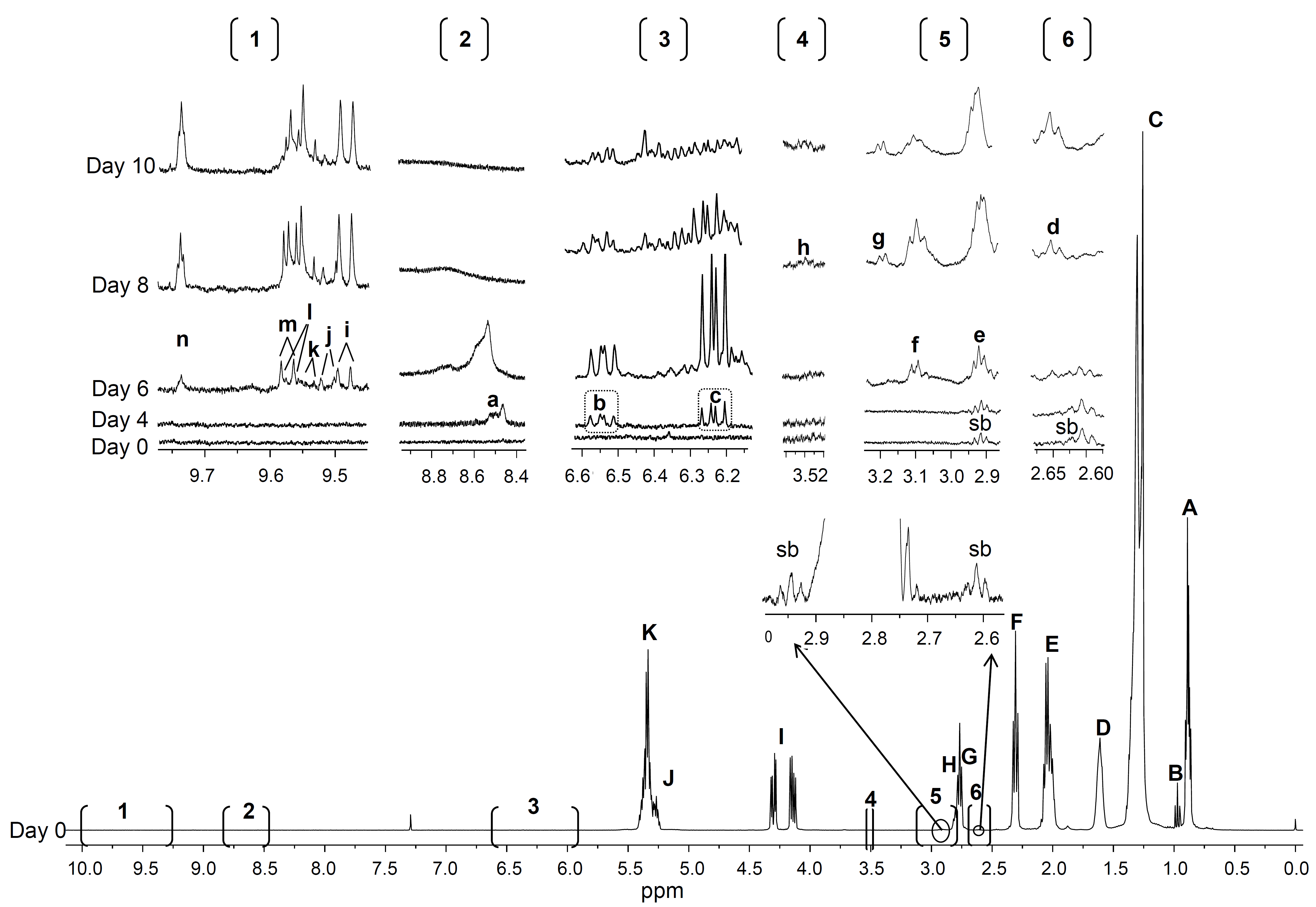


**Figure 1.** ^1^H NMR spectrum of sample VSO before being subjected to the AS process, together with the enlargements of some spectral regions where changes occur throughout time. Letters agree with those in Table S2, considering that “e” includes signals “e1-e6”, “f” signals “f1-f3”, and “h” signals “h1+h2”. The plots corresponding to the same ^1^H NMR spectral region are presented at a fixed value of absolute intensity, for them to be valid for comparative purposes. “sb”: side band of the bis-allylic proton signals (H+G).

**Table S2.** Chemical shifts, multiplicities, and assignments of the ^1^H NMR signals in CDCl_3_ of the main types of triglyceride (TG) protons, and of some oxidation compounds, present in the different soybean oil samples, before and throughout the oxidation process.

| **Signal** | **Chemical shift (ppm)** | **Multi-**  **plicity** | **Functional group** | |
| --- | --- | --- | --- | --- |
|  |  |  | **Type of protons** | **Compound** |
| **Main acyl groups^a^** | | | | |
| **A** | 0.88 | t | -C**H_3_** | Saturated and monounsaturated ω-9 acyl groups |
|  | 0.89 | t | -C**H_3_** | Linoleic acyl groups |
| **B** | 0.97 | t | -C**H_3_** | Linolenic acyl groups |
| **C** | 1.19-1.42 | m* | -(C**H_2_**)_n_- | Acyl groups |
| **D** | 1.61 | m | -OCO-CH_2_-C**H_2_**- | Acyl groups |
| **E** | 1.94-2.14 | m** | -C**H_2_**-CH=CH- | Acyl groups |
| **F** | 2.26-2.36 | dt | -OCO-C**H_2_**- | Acyl groups |
| **G** | 2.77 | t | =HC-C**H_2_**-CH= | Linoleic acyl groups |
| **H** | 2.80 | t | =HC-C**H_2_**-CH= | Linolenic acyl groups |
| **I** | 4.22 | dd,dd | ROC**H_2_**-CH(OR’)-C**H_2_**OR’’ | Glyceryl groups |
| **J** | 5.27 | m | ROCH_2_-C**H**(OR’)-CH_2_OR’’ | Glyceryl groups |
| **K** | 5.28-5.46 | m | -C**H**=C**H**- | Acyl groups |
| **Oxidation compounds** | | | | |
| **Hydroperoxides^b^** | | | | |
| **a** | 8.3-9.0 | bs | -OO**H** | Monohydroperoxide group |
| **Conjugated dienic systems^b^** | | | | |
| **-**  **-**  **-**  **b** | 5.51  5.56  6.00  6.58 | dtm  ddm  ddtd  dddd | -C**H**=C**H**-C**H**=C**H**- | (*Z,E*)-conjugated double bonds associated with hydroperoxy group in octadecadienoic acyl groups |
|  |  |  |  |  |
| **-**  **-**  **-**  **c** | 5.47  5.76  6.06  6.27 | ddm  dtm  ddtd  ddm | -C**H**=C**H**-C**H**=C**H**- | (*E,E*)-conjugated double bonds associated with hydroperoxy group in octadecadienoic acyl groups^c^ |
| **Epoxides** | | | | |
| ***Epoxy-derivatives*** | | | | |
| **d** | 2.63^d^ | m | -C**H**O**H**C- | (*E*)-9,10-epoxystearate |
| **e1** | 2.88^d^ | m | -C**H**O**H**C- | (*Z*)-9,10-epoxystearate |
|  |  |  |  |  |
| **e2** | 2.9^e^ | m | -C**H**O**H**C- | Monoepoxy-octadecenoate groups |
|  |  |  | -C**H**OHC-CH_2_-CHO**H**C- | Diepoxides |
| **e3** | 2.94*** | m | -C**H**O**H**C- | (*Z*)-(12,13)-epoxy-9(*Z*),15(*Z*)-octadecadienoic acid |
| **f1** | 3.10^e^ | m | -CHO**H**C-CH_2_-C**H**OHC- | diepoxides |
| ***Epoxy-keto-derivatives*** | | | | |
| **e4** | 2.89^f^/2.90^g^ | td^h^/m^i^ | -CO-CH**=**CH-CHO**H**C- | (*E*)-9,10-epoxy-13-keto-(*E*)-11-octadecenoate |
| **e5** | 2.91^f^ | td | -C**H**OHC-CH**=**CH-CO- | (*E*)-12,13-epoxy-9-keto-(*E*)-10-octadecenoate |
| **g** | 3.20^f,g^ | dd | -CO-CH**=**CH-C**H**OHC- | (*E*)-9,10-epoxy-13-keto-(*E*)-11-octadecenoate |
|  |  |  | -CHO**H**C-CH**=**CH-CO- | (*E*)-12,13-epoxy-9-keto-(*E*)-10-octadecenoate |
|  |  |  | -C**H**OHC-CH**=**CH-CO- | (*Z*)-12,13-epoxy-9-keto-(*E*)-10-octadecenoate |
|  |  |  | -CO-CH**=**CH-CHO**H**C- | (*Z*)-9,10-epoxy-13-keto-(*E*)-11-octadecenoate |
| **h1** | 3.52^f^ | dd | -CHO**H**C-CH**=**CH-CO- | (*Z*)-12,13-epoxy-9-keto-(*E*)-10-octadecenoate |
| **h2** | 3.53^f^ | dd | -CO-CH**=**CH-C**H**OHC- | (*Z*)-9,10-epoxy-13-keto-(*E*)-11-octadecenoate |
| ***Epoxy-hydroxy-derivatives*** | | | | |
| **e6** | 2.93^h^ | dt | -C**H**OHC-CHOH-CH**=**CH- | *threo*-11-hydroxy-(*E*)-12,13-epoxy-(*Z*)-9-octadecenoate |
| **f2** | 3.09^i^/3.097^j^ | dd | -CHO**H**C-CH**=**CH-CHOH- | 9-hydroxy-(*E*)-12,13-epoxy-(*E*)-10-octadecenoate |
| ***Epoxy-hydroperoxy-derivatives*** | | | | |
| **f3** | 3.11^i^ | dd | -CHO**H**C-CH**=**CH-CHOOH- | 9-hydroperoxy-(*E*)-12,13-epoxy-(*E*)-10-octadecenoate^k^ |
| **Aldehydes** | | | | |
| **i** | 9.49^l^ | d | −C**H**O | (*E*)-2-alkenals |
| **j** | 9.52^l^ | d | −C**H**O | (*E,E)-*2,4-alkadienals |
| **k** | 9.55^l^ | d | −C**H**O | 4,5-epoxy-2-alkenals |
| **l** | 9.57^l^ | d | −C**H**O | 4-hydroxy-(*E*)-2-alkenals |
| **m** | 9.58^l^ | d | −C**H**O | 4-hydroperoxy-(*E*)-2-alkenals |
| **n** | 9.75^l^ | t | -C**H**O | n-alkanals |

t: triplet; m: mutiplet; d: doublet; bs: broad signal; *Overlapping of multiplets of methylenic protons in the different acyl groups either in β-position, or further, in relation to double bonds, or in γ-position, or further, in relation to the carbonyl group; **Overlapping of multiplets of the α-methylenic protons in relation to a single double bond of the different unsaturated acyl groups; ***Assignment made with the aid of standard compounds

^a^Assignments taken from Guillén, M.D.; Ruiz, A. *J. Sci. Food Agric*. **2003***, 83*, 338-346.

^b^Data taken from Goicoechea, E.; Guillén, M.D. *J. Agric. Food Chem*. **2010***, 58*, 6234-6245.

^c^The chemical shifts of the (*Z,E*)- and (*E,Z*)-isomers are practically indistinguishable, according to data from Chan, H.W.S.; Levett, G. *Lipids* **1977***, 12*, 99-104.

^d^Data taken from Du, G.; Tekin, A.; Hammond, E.G.; Woo, L.K. *J. Am. Oil Chem. Soc*. **2004***, 81*, 477-480.

^e^Data taken from Aerts, H.A.J.; Jacobs, P.A. *J. Am. Oil Chem. Soc*. **2004***, 81*, 841-846.

^f^Data taken from Lin, D.; Zhang, J.; Sayre, L.M. *J. Org. Chem*. **2007**, *72*, 9471-9480.

^g^Data taken from Gardner, H.W.; Kleiman, R.; Weisleder, D. *Lipids* **1974***, 9*, 696-706.

^h^Data taken from Garssen, G.J.; Veldink, G.A.; Vliegenthart, J.F.; Boldingh, J. *FEBS J.* **1976***, 62*, 33-36.

^i^Data taken from Gardner, H.W.; Weisleder, D.; Kleiman, R. *Lipids* **1978***, 13*, 246-252.

^j^Data taken from Van Os Cornelis, P.A.; Vliegenthart, J.F.G.; Crawford, C.G.; Gardner, H.W. *Biochim. Biophys. Acta* **1982***, 713*, 173-176.

^k^δ-Ketols (hydroxy-keto-derivatives) could also contribute to this signal (Gardner et al., 1974).

^l^Data taken from Guillén, M.D.; Ruiz, A. *Eur. J. Lipid Sci. Technol*. **2004**, *106*, 680-687.

Determination of the molar percentages of the different kinds of oil acyl groups from ^1^H NMR spectral data

The molar percentages of the several kinds of oil acyl groups were estimated before and throughout the oxidation process by means of the following equations:

| Linolenic (Ln)% = 100(A_H_/3A), | (S1) |
| --- | --- |
| Linoleic (L)% = 100(2A_G_/3A_I_) | (S2) |
| Oleic (O)% (or Monounsaturated%) = 100(A_E_/3A_I_)-Ln%-L%, | (S3) |
| Saturated+modified% = 100-Ln%-L%-O%, | (S4) |

where A_H_ and A_G_ are the areas of the signals of *bis*-allylic protons of linolenic and linoleic groups, respectively (signals “H” and “G” in Table S1). Given that these two signals overlap to a certain extent, the total area corresponding to each of them was calculated using pure trilinolein and trilinolenin (Sigma-Aldrich) as references. A_I_, in turn, is the area of the signal of the protons at *sn*-1 and *sn*-3 positions in the glycerol backbone of triglycerides, while A_E_ corresponds to that of mono-allylic protons (see signals “I” and “E” in Table S1).

Identification and semi-quantification of some compounds by GC/MS

Identification of most of the extracted components was made by comparison of their retention times and mass spectra with those of commercial standards acquired from Sigma-Aldrich (St. Louis, MO, USA) and Larodan Fine Chemicals AB (Malmo, Sweden). Others were identified by matching of their mass spectra with spectra from commercial libraries by more than 85% (W9N08, Wiley ver. 9.0 and NIST ver. 8.0) and also with those obtained from the literature.

Semi-quantification was based on arbitrary units of the mass spectrum base peak area counts divided by 10^5^. The mass spectra base peaks of the several compounds identified, together with their respective molecular weights, are displayed in Table S1.

Standard compounds used for the identification of some compounds by ^1^H NMR

(*E*)-2-Hexenal, (*E*)-2-heptenal, (*E*)-2-decenal, (*E,E*)-2,4-hexadienal, (*E,E*)-2,4-heptadienal, (*E,E*)-2,4-decadienal, 4,5-epoxy-(*E*)-2-decenal and 12,13-epoxy-9(*Z*)-octadecenoic acid methyl ester (isoleukotoxin methyl ester), acquired from Sigma-Aldrich, 4-hydroxy-(*E*)-2-nonenal, 4-hydroperoxy-(*E*)-2-nonenal, *trans*-12,13-epoxy-9-keto-10(*E*)-octadecenoic acid, purchased from Cayman Chemical (Ann Arbor, MI, USA), and *cis-*(12,13)-epoxy-9(*Z)*,15(*Z*)-octadecadienoic acid, acquired from Cymit Quimica (Barcelona, Spain).

Determination from ^1^H NMR spectral data of the concentrations of the various kinds of oxidation products monitored throughout the AS process, expressed as millimoles per mole of triglyceride (mmol/mol TG)

The general equation to carry out this determination was the following:

| [OP] = [(A_OP_/n)/(A_I_/4)]*1000 | (S5) |
| --- | --- |

where A_OP_ is the area of the signal selected for the quantification of each oxidation product (OP) and n the number of protons that generate the signal.

It must be pointed out that for the determination of the so-called major epoxides (see Section 3.2.2.2), signals between 2.87 and 3.17 ppm approximately were considered together. As Table S2 shows, some of the compounds that could give these signals contribute with two protons (signals “e1”, “e2”, “e3”, and “f1”) but others with only one (signals “e4”, “e5”, “e6”, “f2”, and “f3”). However, due to the difficulty of elucidating exactly which of all these types of compounds are present, it was assumed that the signal at approximately 2.9 ppm corresponds mainly to epoxides contributing with two protons and the one at 3.1 ppm to epoxy-compounds contributing with only one. It must also be noticed that to estimate the area of the epoxy-compounds giving signal at 2.9 ppm, it is necessary to subtract the area corresponding to the side-band of the *bis*-allylic protons signal (signals “H” and “G” in Table S2).
